# Additional Interventions to Enhance the Effectiveness of Individual Placement and Support: A Rapid Evidence Assessment

**DOI:** 10.1155/2012/382420

**Published:** 2012-05-22

**Authors:** Naomi Boycott, Justine Schneider, Mary McMurran

**Affiliations:** Institute of Mental Health, University of Nottingham, Sir Colin Campbell Building, Triumph Road, Nottingham NG7 2TU, UK

## Abstract

*Topic*. Additional interventions used to enhance the effectiveness of individual placement and support (IPS). *Aim*. To establish whether additional interventions improve the vocational outcomes of IPS alone for people with severe mental illness. *Method*. A rapid evidence assessment of the literature was conducted for studies where behavioural or psychological interventions have been used to supplement standard IPS. Published and unpublished empirical studies of IPS with additional interventions were considered for inclusion. *Conclusions*. Six published studies were found which compared IPS alone to IPS plus a supplementary intervention. Of these, three used skills training and three used cognitive remediation. The contribution of each discrete intervention is difficult to establish. Some evidence suggests that work-related social skills and cognitive training are effective adjuncts, but this is an area where large RCTs are required to yield conclusive evidence.

## 1. Introduction

Individual placement and support (IPS) has been developed as a standardised approach to supported employment aimed at helping people with severe mental health problems find competitive work [1]. IPS defines the essential principles of “supported employment” programmes, such that these programmes may be rigorously described and studied in different settings across the world, although these terms are often used interchangeably in the literature [1]. IPS directs that supported employment programmes should include seven core elements: (1) a focus on competitive employment, (2) acknowledgement of the individual's personal interests, (3) a rapid job search, (4) integration of mental health and employment services, (5) programme entry based on client choice, (6) time-unlimited client support, and (7) benefits counselling [2]. IPS has proven very effective in improving vocational outcomes amongst people with severe mental illness when compared to other vocational services, with a recent review reporting that 61% of participants enrolled in IPS programmes gained employment, compared to 23% of those on other vocational programmes [3].

IPS does have limitations, however. As the results above would suggest, around 40% of people on IPS programmes do not gain employment despite the support. A second criticism of IPS relates to job tenure of the people employed through these schemes, which tends to be short [4–7]. One review reported average longest job tenure to be 22 weeks [3], while a more recent review of job tenure reported an average length of 9.96 months worked at first job gained through IPS programmes [8]. Therefore, it has been suggested that the next step for development and evaluation is to augment IPS with other interventions which may increase employment rates and improve job tenure [9]. For instance, cognitive skills training programmes may help to overcome the illness-related difficulties in attention, memory and executive functions which can have an impact on vocational outcomes, and rates of competitive employment [10]. Cognitive behavioural therapy (CBT) has also been tried as an adjunct to supported employment in order to manage associated stressors [11, 12]. Murphy et al. [5] proposed adding an educational component to improve a person's work skills.

The aim of this rapid evidence assessment was to identify studies which have sought to improve on the effectiveness of standard IPS by adding a supplementary intervention. The aims are to answer the following questions: (1) what supplementary interventions have been used with IPS? (2) What are the results and what is the quality of those studies? (3) Do supplementary interventions improve employment rates and job tenure compared to IPS alone? (4) Are any supplementary interventions superior to others? Throughout this paper, country or region will be noted, as differences in labour markets, unemployment rates, and welfare systems have been shown to affect the results achievable through IPS programmes [13].

## 2. Method

### 2.1. Rapid Evidence Assessment

 A Rapid evidence assessment provides an overview of existing research on a specific research topic, as well as a simple extraction and synthesis of the relevant data. The methods used to search for and appraise the research are systematic and rigorous, but the depth of the search is limited by the development of search terms and breadth of resources searched. This type of assessment is particularly useful to quickly gather existing evidence in a research area and determine what future research needs to be done [15].

### 2.2. Inclusion and Exclusion Criteria

The search was for empirical studies conducted from 1980 to July 2011. Studies were considered for inclusion if they: involved people with a severe mental illness; indicated the use of IPS or IPS core principles; involved supplementary interventions categorised as skills training, education, cognitive training, or psychotherapeutic techniques. Studies meeting these criteria were only included if the design compared IPS alone with enhanced IPS. Studies involving men and women of any age were included, providing the above criteria were met. The outcomes of interest were competitive employment rates, defined as the cumulative number of people working in a competitive job across the duration of the study, and job tenure, defined as the longest duration worked in the same job over the study. It should be noted that recorded job tenure may be affected by length of followup for each study.

Only publications in English were considered. Book chapters, narratives, and editorials were excluded, as were systematic reviews and meta-analyses, although references were screened for further relevant studies. The reference lists of included studies were also examined for potentially relevant papers. All included papers were evaluated for quality using the Maryland Scientific Methods Scale (SMS) [26], which identifies five categories: (1) correlational studies between an intervention and the outcome; (2) pre- and postdesigns with no control or comparison group; (3) comparison studies, where measures are compared for unmatched comparison groups; (4) as in category (3) but using matched controls or controlling for confounding variables; (5) randomised controlled trials (RCTs), where participants are randomly allocated to experimental or control groups. The scale demonstrates the extent to which threats to internal validity have been controlled for, such as causal direction, confounding factors, chance factors, and selection bias. The strength of the IPS part of the intervention can be ascertained through the application of a standardised fidelity scale, so the use of this scale was also noted in appraising the studies' quality. 

### 2.3. Search Strategy

The search used combined terms from each main concept regarding severe mental illness, IPS, and additional interventions of skills training, cognitive training, education or psychotherapy, (e.g., (severe mental illness or schizophrenia) and (individual placement and support or IPS) and (skills training or social skills training)). The following electronic databases were searched: Embase, Medline, PsycInfo, Cumulative Index to Nursing and Allied Health Literature (CINAHL), the Cochrane Library, ISI Web of Science, Applied Social Sciences Index and Abstracts (ASSIA), and Google Scholar. One reviewer (N. Boycott) screened all titles and abstracts of potentially relevant studies. A second reviewer (A. Akhtar) screened over a third of the titles to establish consistency in application of inclusion criteria. Where the two reviewers did not agree, a third reviewer (J. Schneider) was asked to judge the study's relevance.

### 2.4. Data Analyses

Data regarding employment rates and job tenure were extracted directly from the papers. Odds ratios were calculated where possible using data on participants employed/not employed per experimental group in each study. Where necessary, authors were contacted for further clarification or information.

## 3. Results

**Figure 1:**
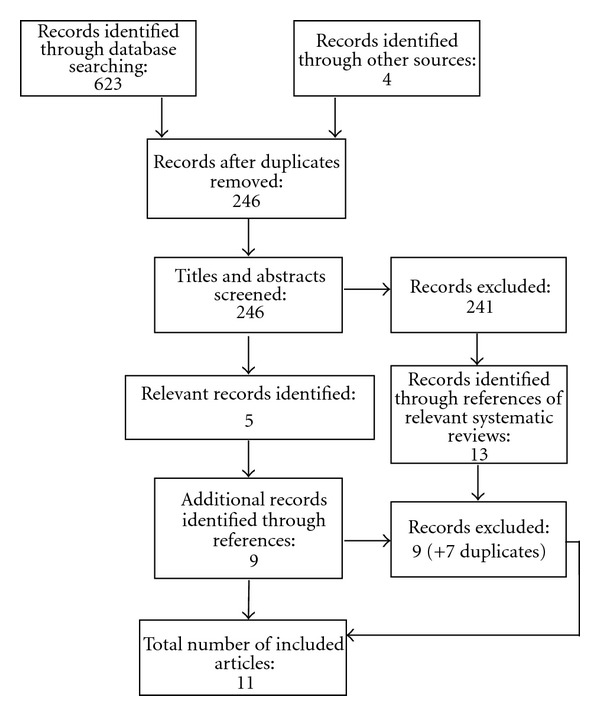
Flowchart of review process. Note: flowchart based on PRISMA flow diagram [14] with amendments.

In total 627 papers were identified and 246 remained after duplicates were removed, of which 241 were excluded at this stage (reasons are given below). References of the 5 included papers and relevant (excluded) systematic reviews were screened for potentially relevant titles, which identified a further 15 records for screening once duplicates had been removed. Of these, 6 further papers met the inclusion criteria. In total, 11 papers were included in the review and 250 papers were excluded (Figure 1). 

### 3.1. Excluded Studies

 Of the 250 papers which were excluded, 108 were not empirical studies: 29 were systematic or literature reviews; 69 were narratives, editorials, or book chapters; 10 were other types of publications, such as practice guidelines, commissioning frameworks, or grant proposals). There were 136 empirical studies of which 81 did not involve IPS or did not specifically assess the effectiveness of IPS, and 39 did involve IPS but without any supplementary intervention. Furthermore, 16 empirical studies were excluded for other reasons: in 8 the population was not people with severe mental illness; 7 were conference abstracts, and 1 did not measure vocational outcomes. The nature of 1 article could not be established, as it was written in Japanese. Finally, 5 papers were excluded as they were not comparative studies of IPS alone versus enhanced IPS (either they did not employ a control group or the control group was not strictly IPS).

### 3.2. Included Studies

 The 11 included articles covered 6 distinct studies, since eight were multiple publications relating to three studies.  Table 1 contains information about the included studies. Three studies used skills training, and three used cognitive remediation. 

### 3.3. What Supplementary Interventions Have Been Used with IPS?

#### 3.3.1. Skills Training

Skills training consisted of two approaches: a work-related social skills package from Hong Kong, which was combined with IPS to create Integrated Supported employment (ISE) [27]; workplace fundamentals training (WFT) [7], which was used in three studies based in the USA. Both programmes were taught using demonstration, role-play, homework assignments, and problem-solving techniques. 

The ISE programme aimed to help participants learn social skills related to retaining a job, such as developing good relationships with colleagues and supervisors, and handling interpersonal conflicts and potentially difficult situations at work. These skills were taught over 10 group sessions, and included training on verbal and nonverbal communication, conversation skills, appearance and assertiveness. Ongoing support was offered in order to generalise the skills learnt into work-life.

The WFT module covered skills such as problem solving in order to cope with stressors, symptoms and health concerns, and learning how to interact successfully with colleagues and supervisors. The number of sessions offered to participants varied by study, although the authors of the module suggested biweekly sessions for 8–12 weeks.

#### 3.3.2. Cognitive Rehabilitation

Three cognitive training techniques were described: neurocognitive enhancement therapy (NET), errorless learning, and the thinking skills for work program [24].

NET consisted of computer-based cognitive training, a social information processing group, and a work feedback group. Cognitive training took place for up to 10 hours per week for a year, with exercises of progressive difficulty targeting attention, language, memory and executive functioning. Participants received specific feedback after job specialists conducted workplace-based observations and interviews with supervisors. In the social information processing groups, participants were taught to give work-based presentations to each other, ask questions, and give feedback.

Few details are available for errorless learning training, however, errorless learning posits that stronger learning can take place through repetitively practising tasks whilst eliminating mistakes during the learning process [28]. Errorless learning has regularly been used with people with learning disabilities [29], dementia [30], and schizophrenia [28].

The thinking skills for work program was conducted by a cognitive specialist in conjunction with the employment specialist and as part of an employment team. A cognitive assessment was completed and information on employment history was gathered, followed by a total of 24 hours of computer exercises covering attention, memory, executive functioning, and other cognitive domains. The specialist also provided cognitive remediation, job search planning, and job support to clients, taking into account the participant's cognitive strengths and the cognitive challenges of the job.

### 3.4. What Are the Results and What is the Quality of the Studies?

All studies included in this paper are reported in Table 1, along with their primary outcomes and relevant statistics.

#### 3.4.1. Skills Training

Four papers from Hong Kong reported RCTs of IPS versus ISE versus traditional vocational rehabilitation (TVR) [6, 16–18]. These four papers cover the same project and have been treated as a single study for the review, referred to as Tsang et al. They reported higher competitive employment and longer job tenure for the ISE group compared to IPS and TVR. It was a well-conducted RCT (SMS 5), with blind assessors, good length of followup, good to fair fidelity to IPS standards, and use of intent-to-treat analyses. However, the selection criteria resulted in more severely impaired individuals being excluded and employment specialists were not blind to group allocation.

 Wallace and Tauber [19] report a small-scale RCT from Santa Barbara, California, of IPS plus WFT versus IPS alone (SMS 5), as yet unpublished [31]. No differences were reported in employment rates between groups, although lower job satisfaction was reported in the control group. The study was limited by the small sample size and the lack of blind assessors, but fidelity to IPS was assessed.

 Mueser et al. [20] also published a small RCT from Dartmouth, New Hampshire, of supported employment plus WFT versus supported employment and treatment as usual (SMS 5). No differences were reported for the time worked or wages earned between the groups. The supported employment programme was not termed “IPS” in this paper, but the authors described monitoring the programme for the standards of supported employment, even though no formal fidelity scale was applied and there was a lack of co-location of vocational and mental health services, which would normally be expected in IPS. However, the authors felt that the programme offered could be considered as IPS (Mueser, personal communication, September 6, 2011). This study was limited by sample size, and only recruited people who had obtained a job in the previous two months, which could introduce bias and limit the generalisability of the findings.

#### 3.4.2. Cognitive Rehabilitation

 Two papers referred to here as Greig et al. describe the same RCT (SMS 5) undertaken in Connecticut, of a vocational programme alone versus a vocational programme plus NET [21, 22]. Participants were found to obtain and maintain higher rates of employment in the group receiving NET compared to controls. The authors of the study regarded the vocational programme to be IPS (Bell, personal communication, August 17, 2011), and reported fair implementation of IPS standards. However, transitional funding was also made available to participants to help them start work more quickly, which is not consistent with the core focus of IPS on competitive employment. This study was fairly well conducted in terms of baseline comparability of groups, using concealed allocation and intent-to-treat analyses. However, assessors and employment specialists were not blind to allocation.

 An abstract by Kern et al. [23] detailed an RCT from Los Angeles, California, of IPS versus IPS plus “errorless learning training” (SMS 5). No differences in employment rates or job tenure were found. No further information could be obtained on this study.

 McGurk and colleagues [24, 25] published two papers about a study in New York which combined the Thinking Skills for Work Program with supported employment versus supported employment alone (SMS 5). Inclusion criteria stated that participants had to have a history of job failure, such as leaving a job or being fired from a job, held for less than three months. Nonetheless, employment rates and earnings were found to be better in the experimental group at 2-3-year followup. Although IPS was not specifically mentioned in the abstract, fidelity to the programme was assessed, with fair to good implementation. This study had an impressive follow-up rate of 100%, although with a modest sample size, and blind assessors were not used.

### 3.5. Do Supplementary Interventions Improve Employment Rates and Job Tenure Better than IPS Alone?

There were four studies which reported cumulative competitive employment rates. Two studies reported mean job tenure. These results are presented in Table 2. The study by Mueser et al. [20] was not included in the table, as participants had to have obtained a job in the previous two months, and therefore cumulative employment rates were 100% for both groups.

Ratios of job tenure for IPS versus enhanced IPS are as follows: 1 : 1.3 (ISE; Tsang et al.) and 1 : 0.9 (NET; Greig et al.). Mueser et al. [20] reported job tenure measured in days for first job obtained: 288.5 versus 331.6 (control versus enhanced IPS, resp.).

Calculated odds ratios varied from 0.5 to 13.71. Apart from the study by Wallace and Tauber [19], odds ratios showed that odds of gaining employment was 2.48 to 13.71 times more likely with enhanced IPS interventions than without. Wallace and Tauber's enhanced intervention (WFT) resulted in odds of employment being half of that in the IPS alone group.

### 3.6. Are Any Supplementary Interventions Superior to Others?

Median rates of competitive employment were 49.85% (mean 52.1%) for IPS or control groups and 76.2% (mean 75.58%) for enhanced IPS groups in this paper. Separating these results according to types of supplementary interventions, for skills training mean employment rates were 86.1% versus 77.95%, and for cognitive training 65.05% versus 26.15% (enhanced IPS versus IPS alone or control, resp.).

Odds ratios for skills training interventions showed improved odds of employment in one study (×3; Tsang et al.), but not in another (×0.5; Wallace and Tauber). Cognitive training interventions showed improved odds of employment by 2.48 to 13.71.

## 4. Discussion

 As the included studies vary in the outcome measures used and level of detail reported, and some studies are only preliminary reports or abstracts, it was difficult to synthesise the results. Averaging across the employment rates reported by four studies, enhanced IPS does appear to produce higher rates of competitive employment compared to IPS or control groups alone. The average enhanced rate of 76% would also appear to be higher than the average IPS employment rates reported in previous reviews [3], and this difference is accentuated when focussing on studies using skills training, where on average the employment rate is 25% higher. In addition, rates were moderately higher than previous reviews for studies involving cognitive training.

Caution needs to be taken when assessing the significance of the improved employment rates in some of these studies, however, as the employment rates reported for the IPS alone groups are substantially lower than would be expected in the Greig and McGurk studies [21, 22, 24, 25]. This may call into question the fidelity of the IPS programmes being used in the control groups and hence alter our conclusions about the apparent effect sizes in these studies. Again, it must be noted that Greig et al. were not using IPS in the strictest sense, as transitional funding was made available, and the selection criteria used in the McGurk studies resulted in only clients with a history of job failure being recruited, which may have adversely affected the employment rates in both groups.

Another important outcome is job tenure, although care must be exercised because results regarding job tenure will be affected by the length of followup for the study. Of the two studies which reported mean job tenure, Tsang et al. [6, 16–18] reported longer tenure than IPS alone in one review [3] but not another [8], and Greig et al. [21, 22] reported tenure as roughly the same as a previous review [3].

The odds of gaining employment were improved by the supplementary interventions in the majority of studies and some potentially promising evidence was found for each type. In particular, cognitive training appeared to increase the probability of gaining employment by a considerable degree. However, for Greig et al. [21, 22], the confidence intervals just overlap 1, meaning that it is possible there is no difference between the odds of employment for the two groups. Also, the poor employment results achieved for the IPS alone groups will have influenced the calculations of odds ratios.

It must be noted that due to the restricted number of studies found in this rapid evidence assessment, and some final reports not being available, the evidence is too limited to draw conclusions on which type of supplementary intervention may be superior in terms of employment rates, job tenure, and odds of gaining employment. However, absence of evidence is not evidence of absence, and there is some suggestion that enhancements of IPS may improve outcomes, as well as ideas for further research and development (see Future Research below).

### 4.1. Clinical Implications

 The hypothesis remains that enhanced IPS may improve the chances of competitive employment beyond that of IPS or vocational services alone. However, the current status of the literature makes it impossible to draw any firm conclusions. In the studies presented in this paper, skills training and cognitive training showed improved employment rates over IPS alone or control groups, and skills training may also improve job tenure.

Although the results are limited and in some cases only preliminary, the most promising results are for work-related social skills training to take place alongside IPS as described by Tsang and colleagues [6, 16–18]. Skills taught through workplace fundamentals training and the Thinking Skills for Work Program may also prove helpful.

Further support or training may be necessary to enable some service users to prepare for and manage a job. The studies may suggest that this training is best provided in conjunction with employment services rather than prior to seeking employment support, when the client is aware of the potential challenges involved in competitive work. Although this may prove more costly in terms of setting up training programmes or providing extra support to clients, it may make IPS programmes more cost-efficient if they are able to produce better results.

### 4.2. Limitations

 The limitations of this review, as in any paper, lie in the search terms and strategy employed. If a study used only the generic term “supported employment” to refer to an intervention that was actually IPS, it might have been omitted, although the co-authors' familiarity with the field guards against this risk. Studies did not always specify how rigidly the IPS criteria were adhered to, so the potency of that part of intervention may sometimes be weak, leading to superior results for the enhanced arm.

Combining employment outcomes to calculate means does not take into account the different contexts in which they were generated. Therefore, the results from individual studies in a given labour market or mental health service context may not generalise to real-world effectiveness in other countries.

As with any review, publication bias may have been an issue, as some studies may have gone unpublished due to negative findings or quality-related issues. However, as described above, authors of the included studies and prominent authors in the area were contacted to identify any unpublished studies on this subject, therefore minimising the risk as much as possible.

### 4.3. Future Research

During the search for relevant articles, five further studies of enhanced IPS were identified, although these were not included as they were not comparative in design and so the contribution of the enhancement could not accurately be judged. Of these studies, one was a further case study of ISE [32], one was an RCT of IPS and WFT versus standard vocational rehabilitation [33], and another study examined assertive community treatment (ACT) and IPS [34]. The latter study consisted of two staff teams: ACT and IPS, who tailored and coordinated care to address individuals' mental health and vocational needs. This coordination of care is now a core element of IPS programmes and so would no longer be considered as an enhancement. Furthermore, one study enhanced IPS with motivational interviewing [35], which aims to resolve individuals' ambivalence for change [36], and one study documented a trainee project, which combined parental support, promotion, and supervision with IPS techniques [37]. Although the latter two studies have not been tested with large-scale RCTs, they may still suggest the potential for future research in this area.

The area of enhanced IPS research and evidence is at an early stage of development and questions about generalisability, effectiveness and costs have yet to be addressed systematically. Now larger, better-controlled studies are needed to test the cost-effectiveness of the most promising approaches. These studies also need to be clear and precise about the terms used, such as IPS or supported employment, and be more descriptive of how faithful their vocational programmes are to the IPS model. A useful next step would be to compare the different adjuncts to each other and IPS alone in order to determine whether one supplementary intervention is superior to others.

## Figures and Tables

**Table 1:** Primary outcomes of included studies.

Supplementary intervention type	Study (Area of origin)	Study design	Experimental condition(s) (*n*)	Control condition (*n*)	Primary outcomes	Statistics
Skills training	Tsang et al. [8, 16–18] (Hong Kong)	RCT	ISE (58)	IPS and TVR (65) (66)	ISE = Higher employment, longer job tenure, fewer interpersonal conflicts than IPS and control at 3 year followup	Employment rate: *χ* ^2^ = 6.78, df = 1, *P* < 0.01 Tenure: *F *= 9.53, df = 4316, *P* < 0.01

	Wallace and Tauber [19] (Santa Barbara, USA)	RCT	IPS + WFT (19)	IPS alone (18)	No differences in earnings or hours worked. More job turnover and less job satisfaction in control group	Employment rate: *χ* ^2^<0.01

	Mueser et al. [20] (Dartmouth, USA)	RCT	SE + WFT (17)	SE (18)	Better knowledge of WFT, no differences on vocational outcomes at 18 months	Tenure: *Z* = 0.30, *P* = 0.53 Effect size: 0.16

Cognitive training	Greig et al. [21, 22] (Connecticut, USA)	RCT	VOC + NET (38)	VOC alone (34)	^#^Higher competitive employment and more hours worked in VOC + NET group	Employment rate: *χ* ^2^ = 3.57, df = 1, *P* < 0.05

	Kern et al. [23] (Los Angeles, USA)	RCT	IPS + errorless learning*	IPS alone*	No differences in job placement or tenure. Twenty people employed	

	McGurk et al. [24, 25] (New York, USA)	RCT	SE + CT (23)	SE (21)	More likely to work in SE + CT group, worked more hours, more jobs and higher earnings at 2-3-year followup	Employment rate: *χ* ^2^ = 18.00, df = 1, *P* < 0.01

Note: results are reported to 2dp. Acronyms: IPS; Individual placement and support; ISE; integrated supported employment; TVR; traditional vocational rehabilitation; WFT; workplace fundamentals Training; SE; supported employment; VOC; vocational programme; NET; neurocognitive enhancement therapy; CT; cognitive training. ^#^Results taken from Bell et al. [22]. *Numbers in each condition unknown, *N* = 45.

**Table 2:** Cumulative employment rates and job tenure across studies.

Supplementary intervention type	Study	Competitive employment-IPS alone or control % (*n*)	Competitive employment-enhanced IPS % (*n*)	Odds ratio 95% CI	Job tenure (weeks)—IPS alone or control Mean (SD)	Job tenure (weeks)—enhanced IPS Mean (SD)	Followup (months)
Skills training	Tsang et al. [8, 16–18]	61.5 (40/65)	82.8 (48/58)	3.00 (1.29, 6.98)	36.17	46.94	39
	Wallace and Tauber [19]	94.4 (17/18)	89.4 (17/19)	0.50 (0.04, 6.05)			18

Cognitive training	*Greig et al. [21, 22]	38.2	60.5	2.48 (0.96, 6.40)	22.05 (15.6)	20.3 (16.0)	24
	McGurk et al. [24, 25]	14.3 (3/21)	69.6 (16/23)	13.71 (3.03, 62.14)			^#^26.8

Tsang et al. results taken from [18]. *Greig et al. results taken from personal communication (Bell, September 20, 2011). ^#^Average length of followup.
